# Comprehensive Analysis of Differentially Expressed Genes and Epigenetic Modification-Related Expression Variation Induced by Saline Stress at Seedling Stage in Fiber and Oil Flax, *Linum usitatissimum* L.

**DOI:** 10.3390/plants11152053

**Published:** 2022-08-05

**Authors:** Ningning Wang, Yujie Lin, Fan Qi, Chunxiao Xiaoyang, Zhanwu Peng, Ying Yu, Yingnan Liu, Jun Zhang, Xin Qi, Michael Deyholos, Jian Zhang

**Affiliations:** 1 Faculty of Agronomy, Jilin Agricultural University, Changchun 131018, China; 2 Information Center, Jilin Agricultural University, Changchun 130000, China; 3 School of Basic Medicine, Guizhou University of Traditional Chinese Medicine, Guiyang 550025, China; 4 Institute of Natural Resource and Ecology, Heilongjiang Academy of Science, Harbin 150040, China; 5 Department of Biology, University of British Columbia Okanagan, Kelowna, BC V1V 1V7, Canada

**Keywords:** flax, saline stress, differentially expressed genes, tissue/organ specificity, epigenetic modification associated

## Abstract

The ability of different germplasm to adapt to a saline–alkali environment is critical to learning about the tolerance mechanism of saline–alkali stress in plants. Flax is an important oil and fiber crop in many countries. However, its molecular tolerance mechanism under saline stress is still not clear. In this study, we studied morphological, physiological characteristics, and gene expression variation in the root and leaf in oil and fiber flax types under saline stress, respectively. Abundant differentially expressed genes (DEGs) induced by saline stress, tissue/organ specificity, and different genotypes involved in plant hormones synthesis and metabolism and transcription factors and epigenetic modifications were detected. The present report provides useful information about the mechanism of flax response to saline stress and could lead to the future elucidation of the specific functions of these genes and help to breed suitable flax varieties for saline/alkaline soil conditions.

## 1. Introduction

Soil salinization has become a severe problem around the world. The area of saline–alkali land accounts for 7% of the world’s aerial land [1]. Understanding the mechanisms of plant response to salinized environmental stress could facilitate crop improvement and allow better agricultural productivity.

Flax, *Linum usitatissimum* L. is a versatile annual plant grown in the north temperate zone. It can be divided into three types: fiber flax, oil flax (linseed), and oil-fiber dual-purpose flax. The seeds of flax are a rich source of α-linolenic acid, which is essential for human health and has been reported to have anti-tumor, antithrombotic, and other functions [2,3]. In addition, lignans, dietary and other functional ingredients in flax seed are widely used in the food industry. Moreover, flax fibers are commonly used in the textile industry. It is therefore important to study flax germplasm resources and their adaptability to grow under different growth environments, including salinized soils.

Previous studies have investigated the salinity response in flax using a variety of techniques [4]. A total of 902 single nucleotide polymorphisms (SNPs) were detected by a Genome-wide Association Study (GWAS) of 200 diverse flax varieties following saline stress [4]. They also found that oil flax was more tolerant than fiber flax under saline stress [4]. Differentially expressed genes were also identified following NaCl treatment in the fiber flax variety Agatha [5]. miRNAs expression was induced by saline, alkaline, and saline–alkaline stresses in the fiber flax variety (Heiya-19) [6]. The differences in response to saline stress between fiber flax and oilseed flax varieties have not been examined.

Saline stress excessively impacts plant growth and development. It induces reactive oxygen species (ROS) and malondialdehyde (MDA) accumulation which damage the structure of the cells in plants. Several classes of enzymes scavenge them in response to such environmental conditions, including catalases (CAT), superoxide dismutase (SOD), peroxidase dismutase (POD), etc. At the same time, small-molecule organic compounds are increased to maintain intracellular water potential, including proline, soluble proteins, sugar, etc., which has been revealed by studies on rice, wheat, cotton, and other plants [7,8,9]. In addition, saline stress can cause disorders in ion balance and the destruction of osmotic pressure in plants. Plant hormones mediate salinity signals to regulate plant growth and development, including abscisic acid (ABA), ethylene, salicylic acid (SA), and jasmonic acid (JA); these are regarded as stress-responsive hormones. Others are classified as growth-promoting hormones, including auxin, gibberellin (GA), cytokinins (CKs), brassinosteroids (BRs), and strigolactones (SLs), which have been widely investigated in saline stress. Meanwhile, hormone-level alterations were associated with a variation in gene expression [10,11,12,13]. TFs can receive a series of signals under stress and activate gene expression to regulate various processes of plant growth and development under adverse stress conditions, including APETALA2/ethylene-responsive factor (AP2/ERF), NAC (NAM, ATAF, and CUC), myeloblastosis (MYB), basic leucine-zipper (bZIP), and basic helix-loop-helix (bHLH), etc. *GsERF6* from soybean was overexpressed in Arabidopsis, which enhanced the plant hormone activity and induced plant tolerance to alkaline stress [14]. The overexpression of *OsbZIP71* upregulates *OsCAT* to scavenge ROS under saline stress [15]. *ChbZIP1*, in the alkaliphilic microalgae *Chlorella* sp. BLD has been reported to increase alkali resistance [16]. *SNAC1* enhances tolerance under drought and saline stress [17]. The overexpression of *GmMYB68* enhances saline–alkali stress in soybean [18]. Alkaline stress induces the expression of *BvWRKY10* and *BvWRKY16* in the shoots and roots, respectively [19]. Ionic transporter genes are activated by *OsbHLH035* to enhance salt tolerance [20].

In this study, oil flax and fiber flax were treated with saline stress to investigate: (1) DEGs induced by saline stress in flax; (2) DEGs in the shoots and roots of flax; (3) the epigenetic modification-related expression analysis of flax under saline stress; (4) the difference in gene expression between oil flax and fiber flax under saline stress. A better understanding of how flax adapts to saline stresses could assist in breeding improved flax varieties for producers in salinized soil conditions.

## 2. Results

### 2.1. Effects of Saline Stress on Physiological Responses in Flax

**Figure 1:**
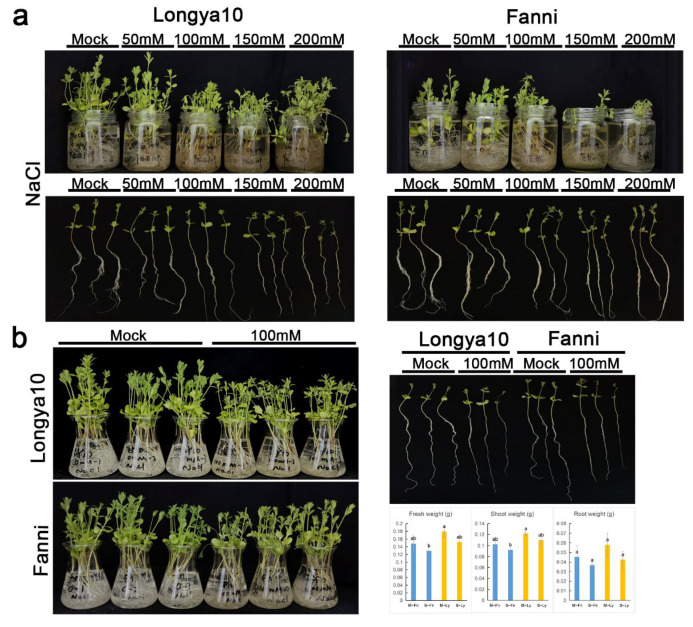
(**a**) Saline stress treatment with 0, 50, 100, 150, and 200 mM NaCl solution for 48 h in Fanni and Longya10. (**b**) Morphological changes in Fanni and Longya10 under 100 mM NaCl solution for 48 h. The mock condition was compared with stress condition as control. M−Fn and M−Ly represent flax growth in the mock condition derived from Fanni and Longya10 plants, respectively. S−Fn and S−Ly represent flax growth in the stress condition derived from Fanni and Longya10 plants, respectively. The values marked a or b between two columns are significantly different at *p* < 0.05.

**Figure 2:**
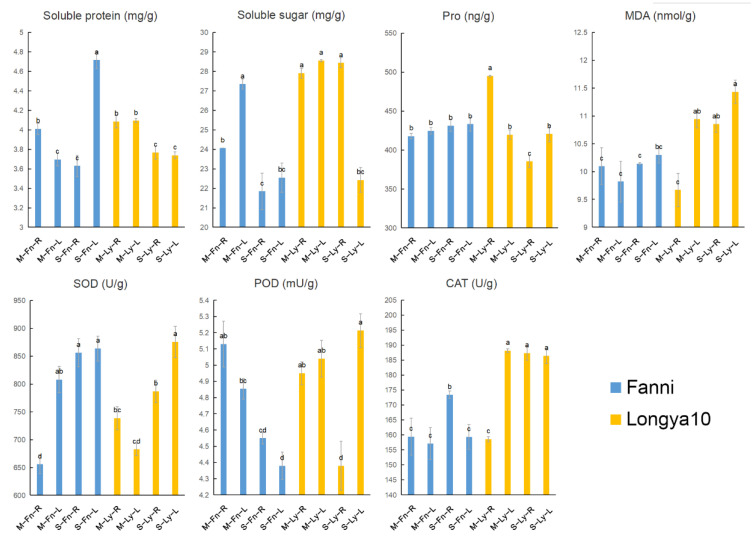
Physiological parameters in the leaf of Fanni and Longya10 under saline stress. The content/activity of the soluble protein, soluble sugar, proline (pro), MDA, SOD, POD, and CAT changes in Fanni and Longya10 under 100 mM NaCl solution for 48 h. M–Fn–R and M–Ly–R represent the root samples derived from Fanni and Longya10 plants, respectively. S–Fn–R and S–Ly–R represent the saline-stress-treated root samples derived from Fanni and Longya10 plants, respectively. M–Fn–L and M–Ly–L represent the leaf samples derived from Fanni and Longya10 plants, respectively. S–Fn–L and S–Ly–L represent the saline-stress-treated leaf samples derived from Fanni and Longya10 plants, respectively. Different letters in the same column represent significant difference (*p* < 0.05).

To explore the macroscopic responses of flax to acute saline stress, we treated the oilseed variety Longya10 and the fiber variety Fanni with different concentrations of NaCl: 0, 50 mM, 100 mM, 150 mM, and 200 mM (Figure 1a). As the concentration of NaCl increased, plant wilting became more severe. We selected an intermediate concentration of 100 mM NaCl for 48 h as our treatment condition for the subsequent analyses and confirmed that all of the plants from both varieties showed similar wilting symptoms and a statistically insignificant decrease in fresh weight (Figure 1b). We next measured the content of soluble protein, soluble sugar, proline, MDA, and the activity of SOD, POD, and CAT in the treated and untreated plants of both varieties (Figure 2). M–Fn–R and M–Ly–R represent the root samples derived from the Fanni and Longya10 plants, respectively. S–Fn–R and S–Ly–R represent the saline-stress-treated root samples derived from the Fanni and Longya10 plants, respectively. M–Fn–L and M–Ly–L represent the leaf samples derived from the Fanni and Longya10 plants, respectively. S–Fn–L and S–Ly–L represent the saline-stress-treated leaf samples derived from the Fanni and Longya10 plants, respectively. These results showed that in response to the stress treatment: (i) soluble protein decreased in the roots of both varieties and in the leaves of Longya10 but markedly increased in the leaves of Fanni; (ii) soluble sugar decreased in the leaves of both varieties and in the roots of Fanni, but was unchanged in the roots of Longya10; (iii) proline decreased significantly only in the roots of Longya10; (iv) MDA increased significantly only in the roots of Longya10; (v) SOD increased significantly only in the roots of Fanni and the leaves of Longya10; (vi) POD decreased in the roots of both varieties and in the leaves of Fanni, but was statistically unchanged in the leaves of Longya10. (vii) the activity of CAT increased in the roots of both varieties but was unchanged in the leaves. Thus, all of the organs and varieties showed physiologically-significant changes in response to the saline treatment we applied, although these responses varied between the organs and varieties.

### 2.2. Effects of Saline Stress on Gene Expression Responses in Flax

**Figure 3:**
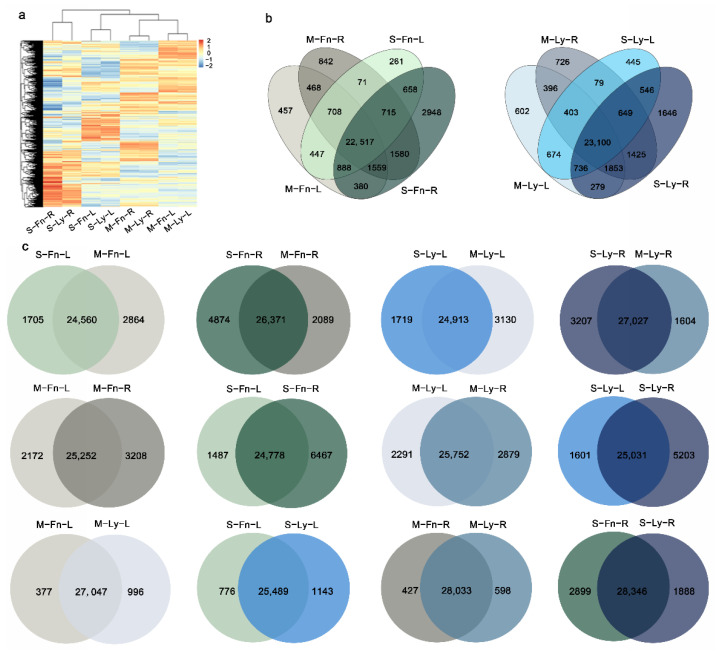
(**a**) Genome-wide gene expression between root and leaf in Fanni and Longya10 under mock or saline stress. Yellow indicates gene expression levels (read counts per million with log2 value). (**b**,**c**) Venn diagram of common and specific gene expression in Fanni and Longya10 under 100 mM NaCl solution. M–Fn–R and M–Ly–R represent the root samples derived from Fanni and Longya10 plants, respectively. S–Fn–R and S–Ly–R represent the saline-stress-treated root samples derived from Fanni and Longya10 plants, respectively. M–Fn–L and M–Ly–L represent the leaf samples derived from Fanni and Longya10 plants, respectively. S–Fn–L and S–Ly–L represent the saline-stress-treated leaf samples derived from Fanni and Longya10 plants, respectively.

To identify the differentially expressed genes that correlated with the stress responses, an RNA-seq of each sample was performed using the Illumina Novaseq platform with pair-end sequencing, which generated from 40 million to 59 million clean reads for each sample (Table S1). The reads were mapped onto the flax reference genome. DESeq2 of the R package was used to normalize the counts of the mapped reads to the gene level. A total of 27, 424 (M–Fn–L), 28,460 (M–Fn–R), 26,265 (S–Fn–L), 31,245 (S–Fn–R), 28,043 (M–Ly–L), 28,631 (M–Ly–R), 26,632 (S–Ly–L), and 30,234 (S–Ly–R) genes were detected by transcriptome analysis. The selected genes were validated by a qRT-PCR, as shown in Supplementary Figure S1, with three biological replicates set for each experiment. The results showed high similarity in the pattern of gene expression between qRT-PCR and RNA-Seq. The genome-wide gene expression pattern was clustered and analyzed by a heatmap (Figure 3a). The co-expression and unique-expression genes were calculated by Venn analysis for Longya10 and Fanni, respectively (Figure 3b). Comparing the different treatments (Figure 3c), there was an 84.31% (24,560/29,129) co-expression of both S–Fn–L and M–Fn–L. In contrast, 5.85% (1705/29,129) and 9.83% (2864/29,129) of the genes were uniquely expressed in S–Fn–L compared to M–Fn–L and vice versa. In all of the comparisons, the number of expressed genes in the roots was more than the number of the expressed genes in the leaves. As identified in the comparison of different organs, the percentage of unique gene expression was higher in the roots derived from Fanni and longya10 following saline stress. Meanwhile, the different number of expressed genes indicated that the mechanism of response to saline stress might be similar but somewhat different between the two tissues or between the two genotypes. Therefore, it was necessary to explore the differentially expressed genes between the stressed and control root and leaf in fiber flax and oil flax, respectively.

### 2.3. Differentially Expressed Genes Induced by Saline Stress in Flax

**Figure 4:**
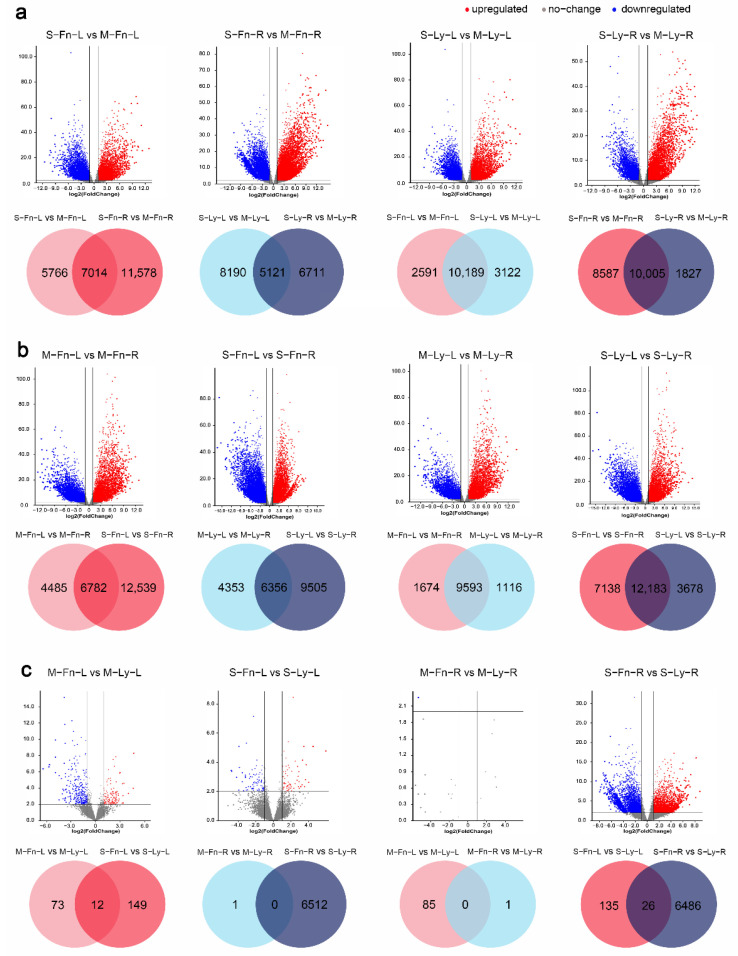
Differentially expressed genes (DEGs) were induced by (**a**) saline stress, (**b**) different organs, or (**c**) different genotype in Fanni and Longya10. The upregulated DEGs were marked with red spots, and the downregulated DEGs were marked with blue spots on the volcano map, respectively. Common and unique DEGs were calculated by Venn analysis. M–Fn–R and M–Ly–R represent the root samples derived from Fanni and Longya10 plants, respectively. S–Fn–R and S–Ly–R represent the saline-stress-treated root samples derived from Fanni and Longya10 plants, respectively. M–Fn–L and M–Ly–L represent the leaf samples derived from Fanni and Longya10 plants, respectively. S–Fn–L and S–Ly–L represent the saline-stress-treated leaf samples derived from Fanni and Longya10 plants, respectively.

To explore the differences in the transcriptional responses to saline stress, differentially expressed genes (DEGs) were identified between the two samples (>2 folds, *p* < 0.05) (Figure 4). Under saline stress (Figure 4a), 5525 upregulated genes and 7255 downregulated genes were induced by saline stress in the leaves of Fanni (comparison S–Fn–L with M–Fn–L), whereas 9817 upregulated genes and 8775 downregulated genes were induced by saline stress in the roots of Fanni (comparison S–Fn–R with M–Fn–R). The same trends were detected in Longya10, with 5720 upregulated genes and 7591 downregulated genes induced by saline stress in the leaves of Longya10 (comparison S–Ly–L with M–Ly–L), and 6647 genes were upregulated, and 5185 downregulated genes were induced by saline stress in the roots of Longya10 (comparison S–Ly–R with M–Ly–R). With Fanni, the number of DEGs was largest when the stressed-roots and mock-roots were compared.

We used Venn diagrams to compare the DEGs identified in various comparisons (Figure 4). These diagrams showed that there were 7014 co-DEGs between “S–Fn–L vs. M–Fn–L” and “S–Fn–R vs. M–Fn–R,” 5766 unique DEGs of S–Fn–L vs. M–Fn–L, and 11,578 unique DEGs of S–Fn–R vs. M–Fn–R, respectively. Similar results were observed in Longya10 by comparing “S–Ly–L vs. M–Ly–L” and “S–Ly–R vs. M–Ly–R”. These patterns suggest leaves and roots respond to saline stress by different mechanisms. In leaves, there were 10,819 co-DEGs in comparison of “S–Fn–L vs. M–Fn–L” and “S–Ly–L vs. M–Ly–L”, 2591 unique DEGs of S–Fn–L vs. M–Fn–L, and 3122 unique-DEGs of S–Ly–L vs. M–Ly–L, respectively. In roots, there were 10,005 co-DEGs between “S–Fn–R vs. M–Fn–R” and “S–Ly–R vs. M–Ly–R”, 8587 unique-DEGs of S–Fn–R vs. M–Fn–R, and 1827 unique-DEGs of S–Ly–R vs. M–Ly–R, respectively, which confirmed the results that more genes were induced by saline stress in the roots of Fanni. When the comparisons were made first between different organs (Figure 4b): there were 5424 upregulated genes and 5843 downregulated genes (comparison M–Fn–L with M–Fn–R), the number of up-or down-regulated genes was changed to 8182 and 11,139 after saline stress (comparison S–Fn–L with S–Fn–R), respectively. This indicates that more genes were induced to adapt saline stress. The comparison of Longya10 identified the results. There were 6782 co-DEGs between “M–Fn–L vs. M–Fn–R” and “S–Fn–L vs. S–Fn–R,” 4485 unique DEGs of M–Fn–L vs. M–Fn–R, and 12,539 unique DEGs of S–Fn–L vs. S–Fn–R, respectively. Similar results were observed in Longya10 by comparing “M–Ly–L vs. M–Ly–R” and “S–Ly–L vs. S–Ly–R”. Both results confirmed that more DEGs were induced by saline stress in flax. Under differential cultivars (Figure 4c), most DEGs were clustered on comparison S–Fn–R with S–Ly–R (2926 upregulated genes and 3586 downregulated genes), indicating a differential mechanism response to saline stress between Fanni-roots and Longya10-roots.

**Figure 5:**
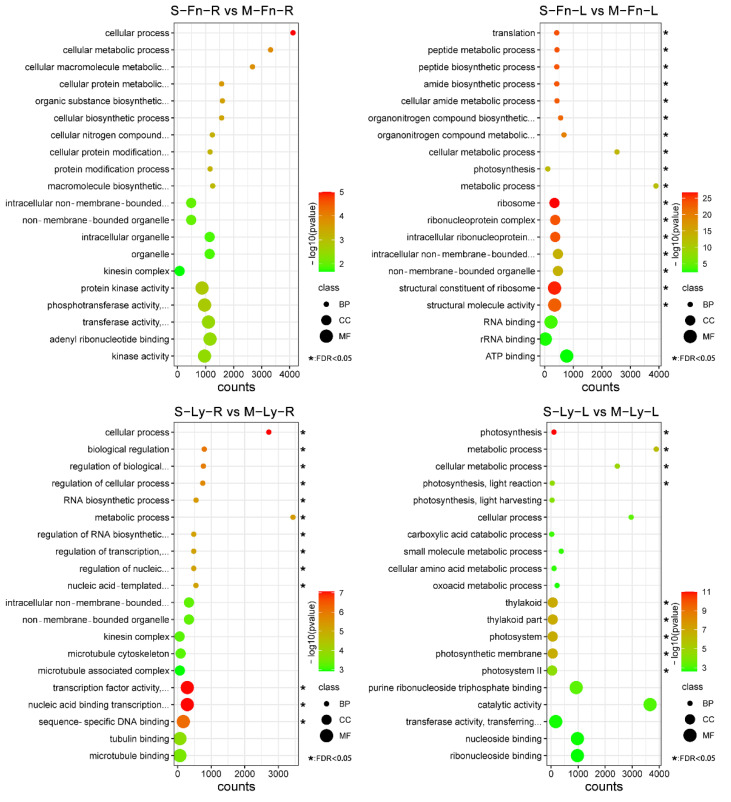
GO classification and enrichment analysis of differentially expressed genes (DEGs) between root and leaf in Fanni and Longya10 under 100 mM NaCl solution. M–Fn–R and M–Ly–R represent the root samples derived from Fanni and Longya10 plants, respectively. S–Fn–R and S–Ly–R represent the saline-stress-treated root samples derived from Fanni and Longya10 plants, respectively. M–Fn–L and M–Ly–L represent the leaf samples derived from Fanni and Longya10 plants, respectively. S–Fn–L and S–Ly–L represent the saline-stress-treated leaf samples derived from Fanni and Longya10 plants, respectively.

For a comparative analysis of the effect of saline stress on flax, we selected the differentially expressed genes under saline stress for further study, including S–Fn–R vs. M–Fn–R, S–Fn–L vs. M–Fn–L, S–Ly–R vs. M–Ly–R, and S–Ly–L vs. M–Ly–L. DEGs were enriched in differential gene ontology pathways. We analyzed the results from agriGO with significant differences (Figure 5, Table S2). Interestingly, the difference in the GO categories between “S–Fn–L vs. M–Fn–L” and “S–Ly–L vs. M–Ly–L”, indicated that the mechanism responding to saline stress was different between Fanni and Longya10. Moreover, the difference in the GO categories between “S–Ly–L vs. M–Ly–L” and “S–Ly–R vs. M–Ly–R”, indicated that the mechanism responding to saline stress was different between the leaves and roots of flax.

### 2.4. Effects of Saline Stress on Plant Hormone-Related Gene Expression in Flax

**Figure 6:**
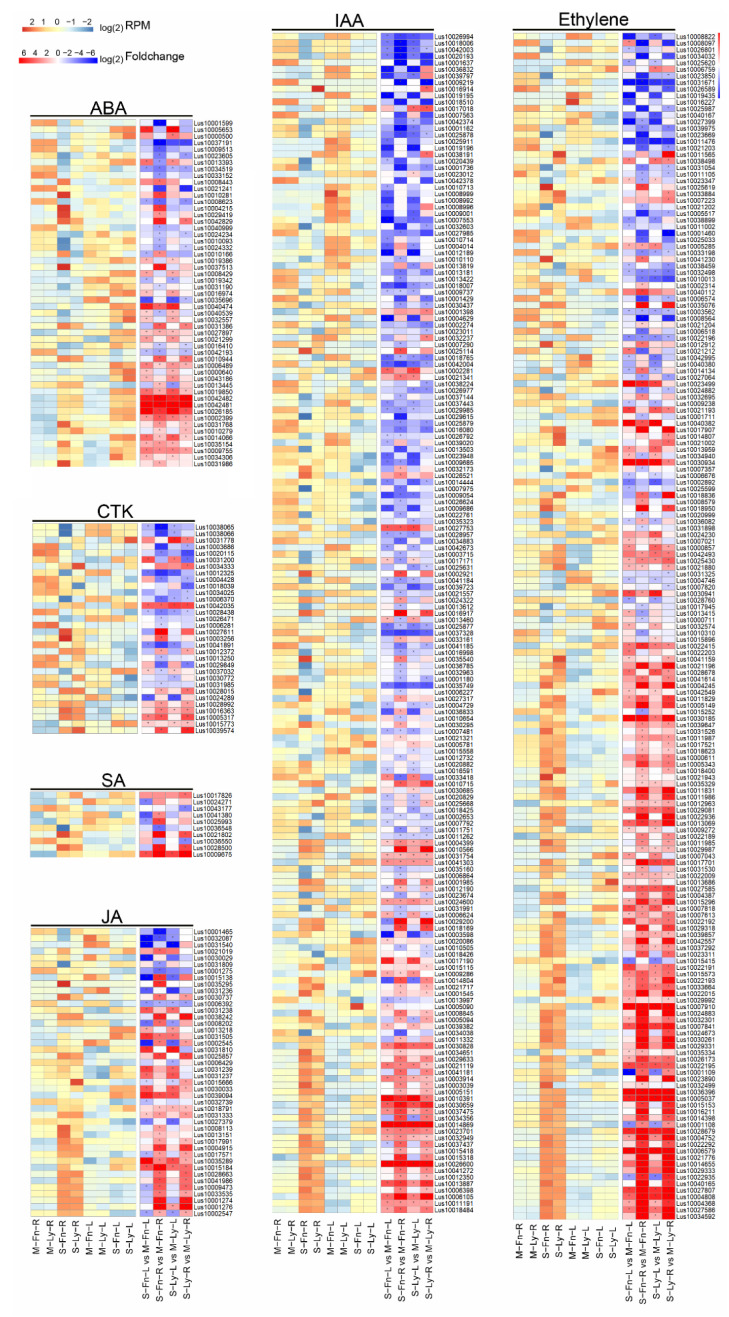
Expression of genes related to plant hormones between root and leaf in Fanni and Longya10 under saline stress. M–Fn–R and M–Ly–R represent the root samples derived from Fanni and Longya10 plants, respectively. S–Fn–R and S–Ly–R represent the saline-stress-treated root samples derived from Fanni and Longya10 plants, respectively. M–Fn–L and M–Ly–L represent the leaf samples derived from Fanni and Longya10 plants, respectively. S–Fn–L and S–Ly–L represent the saline-stress-treated leaf samples derived from Fanni and Longya10 plants, respectively.

Plant hormones have been widely investigated as regulators of plant growth adaptation under saline stress. Our results analyzed plant hormone-related gene expression in fiber flax and oil flax. Using MapMan software, genome-wide DEGs induced by saline stress were analyzed with a focus on plant hormones. The results showed that the ABA, SA, JA, and ethylene–related genes significantly varied with saline stress in flax (Figure 6, Table S3). In particular, the hormone-related saline-stress DEGs differed between the leaves and roots in flax.

### 2.5. Effects of Saline Stress on Transcription Factor-Related Gene Expression in Flax

**Figure 7:**
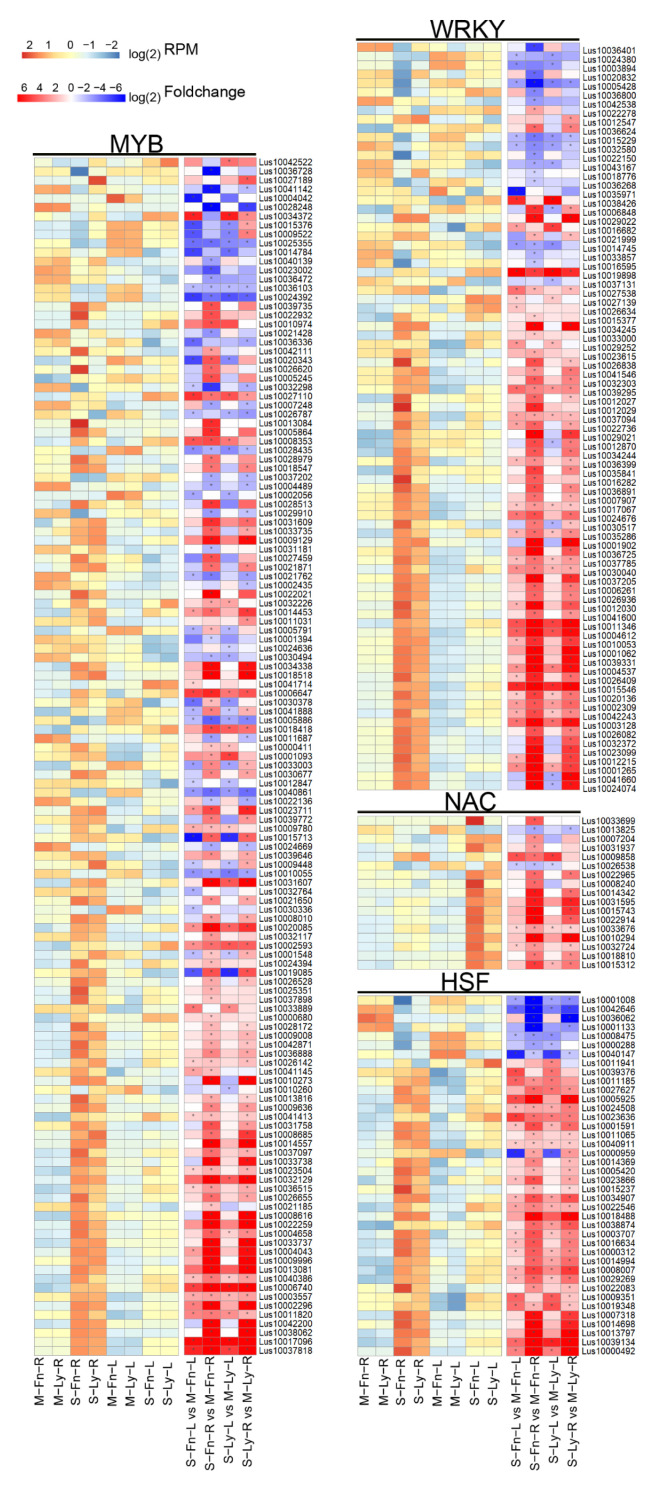
Expression of genes related to TFs between root and leaf in Fanni and Longya10 under saline stress. M–Fn–R and M–Ly–R represent the root samples derived from Fanni and Longya10 plants, respectively. S–Fn–R and S–Ly–R represent the saline-stress-treated root samples derived from Fanni and Longya10 plants, respectively. M–Fn–L and M–Ly–L represent the leaf samples derived from Fanni and Longya10 plants, respectively. S–Fn–L and S–Ly–L represent the saline-stress-treated leaf samples derived from Fanni and Longya10 plants, respectively.

Transcription factors play an essential role in regulating transcriptional processes across plant genomes under abiotic stress. The alteration of transcription factor-related gene expression is associated with the activated transduction of the signals to adapt to environmental change, resulting in plant salt stress tolerance. In our results, more than thirty transcription factor families with DEGs were involved in saline-stress responses, as revealed by MapMan analysis. Interestingly, most of these genes were upregulated by the induction of saline stress in fiber flax and oil flax, such as WRKY transcription factor–related genes (Figure 7, Table S4). Most DEGs were upregulated in flax by saline stress, especially in root tissue. WKRY transcription factor–related gene expression differed between fiber flax and oil flax to respond to salt stress (Figure 7, Table S4). Our results indicated that the WRKY transcription factor family, MYB transcription factor family, NAC transcription factor family, and HSF transcription factor family were involved in saline-stress responses in fiber flax and oil flax.

### 2.6. Effects of Saline Stress on Ionic Transport-Related Gene Expression in Flax

**Figure 8:**
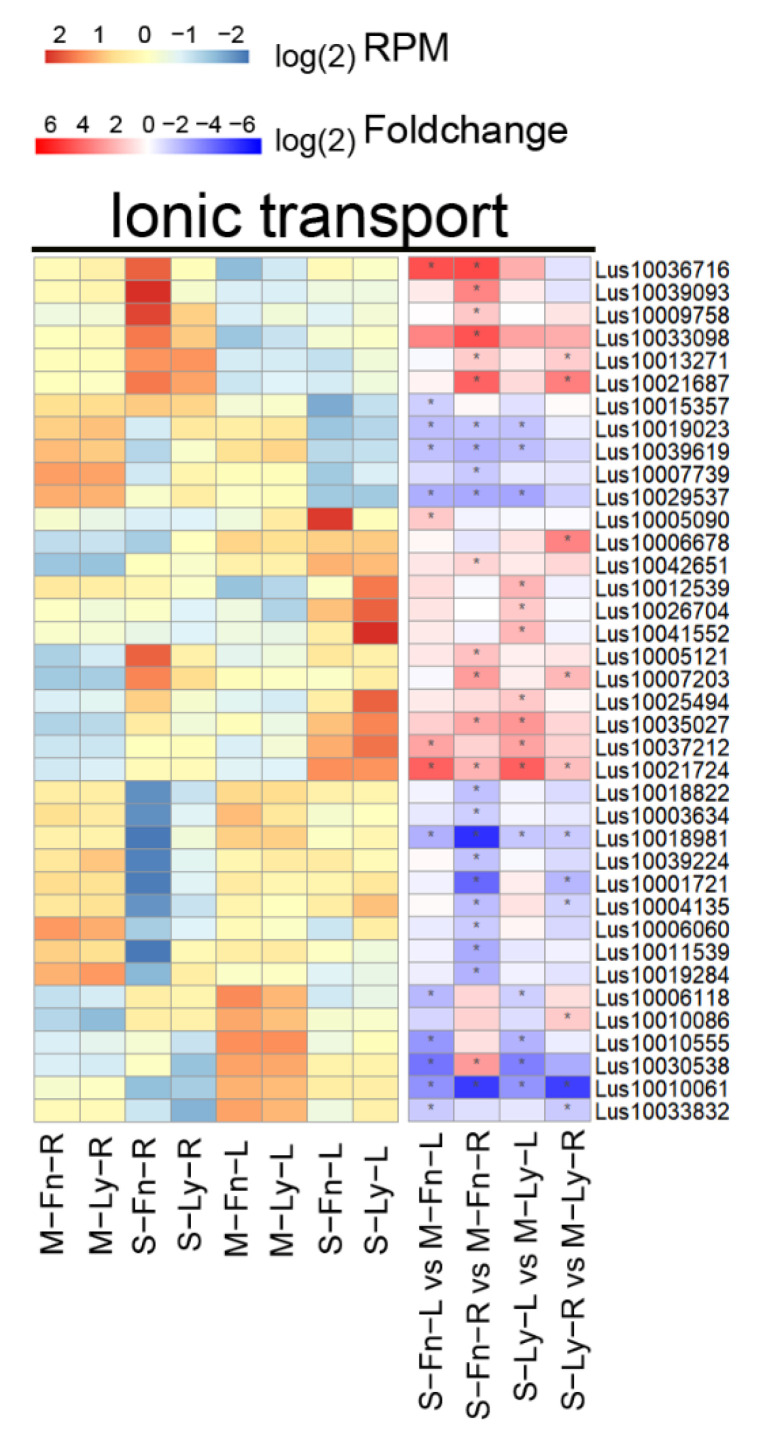
Expression of genes related to ionic transport between root and leaf in Fanni and Longya10 under saline stress. M–Fn–R and M–Ly–R represent the root samples derived from Fanni and Longya10 plants, respectively. S–Fn–R and S–Ly–R represent the saline-stress-treated root samples derived from Fanni and Longya10 plants, respectively. M–Fn–L and M–Ly–L represent the leaf samples derived from Fanni and Longya10 plants, respectively. S–Fn–L and S–Ly–L represent the saline-stress-treated leaf samples derived from Fanni and Longya10 plants, respectively.

In our results, we detected DEGs induced by saline stress that are associated with ionic transport. The genes significantly varied between the treatment and control in flax (Figure 8, Table S5). A comparison of the gene expression between the different organs showed that the tissue of the roots that responded to saline stress was more sensitive than the leaves. Additionally, in a comparison of saline stress with the control, the genes expressed exhibited genotypic specificity. In other words, the gene expression induced by saline stress differed between fiber flax and oil flax. The gene expression levels were clearly different when comparing S–Fn–R vs. S–Ly–R and S–Fn–L vs. S–Ly–L.

### 2.7. Effects of Saline Stress on Epigenetic Modification-Related Gene Expression in Flax

**Figure 9:**
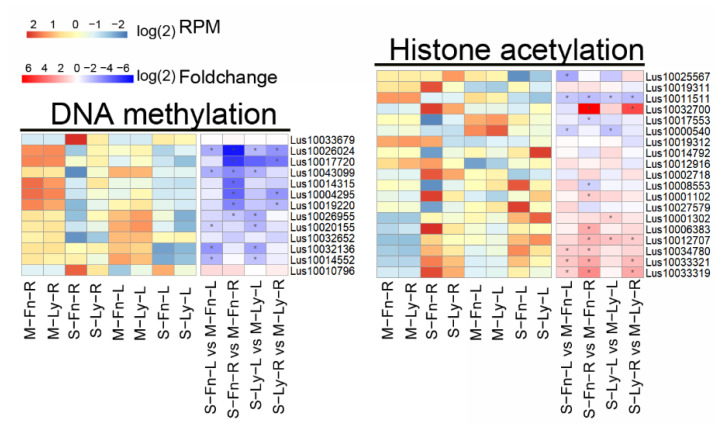
Expression of genes related to epigenetic modification between root and leaf in Fanni and Longya10 under saline stress. M–Fn–R and M–Ly–R represent the root samples derived from Fanni and Longya10 plants, respectively. S–Fn–R and S–Ly–R represent the saline-stress-treated root samples derived from Fanni and Longya10 plants, respectively. M–Fn–L and M–Ly–L represent the leaf samples derived from Fanni and Longya10 plants, respectively. S–Fn–L and S–Ly–L represent the saline-stress-treated leaf samples derived from Fanni and Longya10 plants, respectively.

DNA methylation and histone acetylation are two significant mechanisms of epigenetic modification, which can be induced by abiotic stress. In flax, we detected DEGs associated with saline stress through comparative analysis with DNA methylation and histone acetylation based on MapMan software (Figure 9, Table S6); the results showed that the possible genes related to DNA methylation were downregulated; in contrast, most of the potential genes related to histone acetylation were upregulated when induced by saline stress.

## 3. Discussion

Flax is an important alternative crop in rotations, and flax with improved tolerance to stress can be used to expand cultivation into currently undeveloped and marginal lands. It is therefore desirable to develop elite flax germplasm with a tolerance for different stresses, including saline stress. This study reported on the morphological, physiological, and transcriptome changes induced by saline stress in flax. The expression of several genes related to hormone metabolism, TF activity, ionic transport, and epigenetic modification were induced by saline stress, and these responses differed between the roots and leaves, between fiber flax and oil flax. This study provided additional genetic information on how flax responds to saline stress.

In this study, tissue specificity had a significant effect on saline-stress-induced gene expression; in particular, the roots exhibited genotype-specific responses that differed between Fanni and Longya10. A combined morphological and physiological parameter analysis suggested that the flax’s root is more sensitive than the leaves under saline stress, which is consistent with the results from other species [21,22,23]. Our results demonstrated that the greater tolerance in roots compared to leaves is due to a well-recognized temporal effect in salinity studies wherein root tissues are directly exposed to salt, while leaves initially experience indirect stress due to low water potential and only later do the leaves accumulate enough salt to experience a direct salt effect [24]. A significant number of DEGs also responded in a tissue-specific manner between the leaves and roots. Meanwhile, the number of DEGs increased after saline stress, especially in Fanni, which indicated the mechanisms of response to saline stress between the leaves and roots could be unique in Fanni. Between Fanni and Longya10, there were fewer DEGs under mock conditions in contrast to increasing DEGs in roots after saline stress; this led us to assume the mechanisms of these two types of flax roots in response to saline stress might be different.

The results of the GO enrichment showed that when comparing the saline-treated and control plants, the photosynthesis-related pathways were significantly enriched in the leaves of Fanni and Longya10 at *p* < 0.05. An analysis of the expression of these genes revealed that they were downregulated under salt stress (Supplementary Figure S2) and combined with the wilting of the shoots of flax exhibited in the phenotype. It suggests that salt stress may affect plant growth by affecting water usage in the shoots and thus disrupting the photosynthetic system. Previous studies have shown that photosynthesis is impaired in plants under salt stress [25,26,27], which is in agreement with this study. Polyamines, amides, and biogenic amines were significantly enriched in the leaves and roots of Fanni and Longya10, further validating that those polyamines play an essential role in the plant’s response to abiotic stress [28]. In addition, epigenetic-modifying-related pathways, including methylation and protein modification, were also significantly enriched, and epi-modification was widely shown to play an essential role in stress [29,30].

The gene expression related to the plant hormones (ABA, IAA, CTK, SA, JA, and ethylene) in flax was investigated in the present study. Root growth was inhibited by saline stress, which is associated with reduced auxin accumulation and the repressed gene expression of auxin receptors [13,31]. This suggested that maintaining low auxin signaling affects how plants respond to regulate plant growth adaptation. In our study, most of the DEGs involved in IAA–related genes occurred in the roots in flax after saline stress. The number of DEGs in Fanni’s root was higher than in Longya10′s. TRANSPORT INHIBITOR RESPONSE 1 (*TIR1*) and AUXIN SIGNALLING F-BOX (*AFB*) F-box proteins are critical for receptors in auxin [13]; the expression of the auxin receptor-encoding genes *TIR1* and *AFB2* was downregulated by salt stress [32]. We found that the gene *Lus10018425* was highly similar to *TIR1,* and the gene of *Lus10011262*, *Lus10020086*, and *Lus10018426* was highly similar to *AFB3*, respectively. Most of these genes were downregulated in the flax that had been treated with saline stress. IAA-LEUCINE RESISTANTLIKE2 (*ILL2*) and IAA-ALANINE RESISTANT3 (*IAR3*) are IAA-amino acid conjugate hydrolases that release free IAA by cleaving IAA-amino conjugates [33]. *Lus10029200* (*ILL2*) and *Lus10018169* (*ILL6*) were upregulated in the root after saline stress, whereas the genes *Lus10010715*, *Lus10030828*, and *Lus10030659* were moderately similar to *IAR3* and were upregulated in both the root and leaf after saline stress. Additionally, the expression of IAA-amino synthase genes (GH3 family protein) was altered after saline stress [34,35]. We found *Lus10039797*, *Lus10018510*, *Lus10039723*, *Lus10003598*, *Lus10014804*, *Lus10003039*, *Lus10005151*, *Lus10010391*, *Lus10014869*, *Lus10026600*, and *Lus10013887* were highly similar to *GH3.1*, *GH3.5*, and *GH3.6*; most of them were upregulated in root and leaf after saline stress and among them, Fanni’s root showed the highest expression level. The genes *SAUR36* and *SAUR32* in apple rootstocks’ roots was significantly induced by stress [36]. We found that 46 DEGs were related to the SAUR-like auxin-responsive protein family, in which most of the genes were downregulated in the leaf and upregulated in the root after saline stress. Our results suggested that *SAUR* genes respond to saline stress differed between the root and leaf in flax. ABA is an essential plant hormone that responds to saline stress, the hormone levels increased rapidly and it was associated with gene expression variation. ABA biosynthesis genes, such as 9-cisepoxycarotenoid dioxygenase (*NCED3* and *NCED*5) and abscisic aldehyde oxidase 3 (*AAO3*), were upregulated under moderate dehydration stress in Arabidopsis [37]. The gene *Lus10026185* showed high similarity to *NCED3* and was upregulated in the roots and leaves of flax after saline stress. There were four *AAO2* genes (*Lus10013393*, *Lus10019386*, *Lus10008429*, and *Lus10040474*) with high expression alterations that were detected nor *AAO3*. Most of these genes were upregulated in the leaves of flax after saline stress. At the same time, gene *Lus10031768*, which is nearly identical to *AT1G16540*–*ABA3* appeared to be a positive regulator in ABA synthesis [38] and was upregulated in the root of flax after saline stress. Additionally, it has been reported that ABA-responsive element (ABRE)-binding protein/ABRE-binding factor (*AREB*/*ABF*) transcription factors (bZIP family members) regulate stomatal closure in response to osmotic stress in plants [13,39]. There were six *ABF* genes (*Lus10027897*, *Lus10006489*, *Lus10019850*, *Lus10002399*, *Lus10014066*, and *Lus10009755*) were upregulated in the root and leaf of flax after saline stress. Ethylene levels increase in the plant to respond to the saline stress [11,40]. Its precursor 1-aminocyclopropane-1-carboxylic acid (ACC) synthase (*ACS*) genes are upregulated under dehydration stress in Arabidopsis [37]. Most *ACS* genes appeared upregulated (*ACS1*, *ACS6*, *ACS8*, and *ACS10*) in our study. CK levels have been reported to be reduced in order to enhance tolerance to salt stress in plants [41,42]. The knockout of isopentenyl transferase (*IPT*) or the overexpression of CK oxidases/dehydrogenases (*CKXs*) would reduce CK levels as well [43]. We found that the genes *Lus10004428* and *Lus10034025*, which are similar to *IPT5*, were downregulated in flax root; *Lus10012372* and *Lus10028015*, which are similar to *IPT3*, were downregulated in flax leaf and upregulated in the root. There were 13 genes similar to *CKXs* in flax, and most of them were upregulated in the root and leaf. JA has been confirmed it respond to saline stress in the plant [44,45,46]. The level of JA increased, and the signal was activated under stress. It has been reported that lipoxygenase (*LOX2*) and allene oxide cyclase (*AOC2*), which showed a >2-fold increase in expression levels at 48 h after dehydration stress in Arabidopsis [37]. We found that most LOX genes were upregulated in flax, AOC genes were upregulated in the root, and downregulated in the leaf. Our results supported the notion that JA mediates plant growth response to salt stress in a tissue/organ-dependent manner [13]. It has also been reported that SA has a dose effect under saline stress [13,47,48]; we found several genes with a significant difference after saline stress in flax, including six genes belonging to the S-adenosyl-L-methionine-dependent methyltransferases superfamily protein and most of them were upregulated under stress.

TFs receive stress signals and bind to cis-regulatory sequences to regulate downstream gene expression [49,50,51,52]. In soybean, *GmMYB68* overexpression enhanced salt–alkali resistance in transgenic plants [18]. In Alfalfa, RNA-seq reveals that MYBs participate in MYB transcriptional activation, and *MsMYB4* contributes to salinity stress [53]. Moreover, *MsWRKY11* is involved in increasing the contents of chlorophyll, proline, soluble sugar, SOD, and CAT and reducing the contents of MDA and ROS. In our results, the gene *Lus10026936* (similar to *MYB11*) was upregulated in the root of flax, while the range of CAT was increased in the root after saline stress. This indicates that *MYB11* expression in flax is associated with CAT content in response to saline stress. In addition, we found that most of the genes related to MYB domain protein were upregulated in flax after saline stress, including *Lus10011820* (similar to *MYB15*), *Lus10002296* (similar to *MYB20*), and *Lus10037818* (similar to *MYB108*), etc. In contrast, the genes *Lus10028435* (similar to *MYB7*) and *Lus10036103* (similar to *MYB65*) were downregulated in flax. The WRKY gene family contains specific TFs that respond to abiotic stress [7,54]. We found that most of the genes related to WRKY DNA-binding proteins were upregulated in flax after saline stress, such as *Lus10020136* (similar to *WRKY11*), *Lus10002309* (similar to *WRKY40*), *Lus10042243* (similar to *WRKY26*), and *Lus10003128* (similar to *WRKY75*), etc. On the contrary, the genes of *Lus10015229* (similar to *WRKY65*) and *Lus10032580* (similar to *WRKY32*) were downregulated in flax. NAC transcription factor family genes are regulated and induced by salinity and alkali stress [55,56]. *Lus10033676* (similar to *NAC50*) and *Lus10015312* (similar to *NAC76*) were upregulated in flax. Interestingly, most of the DEGs related to TFs were enriched in the root more than that in the leaf, particularly gene expression in the root of Fanni after saline stress. This may suggest that flax TFs respond to saline stress in an organ-dependent and genotype-dependent manner.

Heat shock transcription factor (HSF) binds to the highly conserved heat shock element (HSE) 5 ‘- nGAAn-3′ to aggregate with TFs to regulate gene expression in the process while the plant ares under abiotic stress [57]. In flax, we assayed 30 putative HSF genes, 27 of them (90%) were upregulated in the root and leaf after saline stress, suggesting that HSF genes may play an essential role in the response of flax to saline stress.

Epigenetic modification regulates gene expression under abiotic stress, mainly through DNA methylation and histone acetylation [58,59,60,61]. Previous reports indicated that DNA methylation could be induced by abiotic/biotic stress to regulate gene expression and transposon mobilization in plants [62,63]. DNA methyltransferases such as *DRM1*, *DRM2*, *MET1*, and *CMT3* are essential for establishing de novo methylation and maintenance methylation in the plants [64]. We assayed 12 putative DEGs similar to *MET1*, *CMT3*, and *DRM2*; most of them were downregulated in flax root and leaf after saline stress. Additionally, there were 21 putative DEGs related to histone acetylation; most of them were upregulated in flax root and leaf after saline stress. The DEGs associated with an epigenetic modification in root and leaf were different, suggesting flax plant genomes might be subject to epigenetic change with tissue/organ specificity when flax is under saline stress. These findings will enhance our understanding of flax gene expression regulatory mechanisms under saline stress and subsequently assist in the breeding of improved flax varieties to face the growing problem of saline/alkaline soil conditions.

## 4. Materials and Methods

### 4.1. Plant Materials and Treatment

Fanni is a type of fiber flax, whereas Longya10 is a type of oil flax (i.e., linseed). The seeds were thoroughly washed with distilled water and then transferred to a greenhouse under a 26/18 °C 16/8 h light/dark regime, which were planted in the soil (mixed with vermiculite 1:1) until the seedlings of the flax grew to 8–10 cm and were then rinsed to remove the soil. They were then divided into five groups and placed into the flask (the volume is 200 mL), which responded to differential concentrations of NaCl with distilled water. The concentrations of the NaCl solution were 0, 50 mM, 100 mM, 150 mM, and 200 mM. All of the experimental groups were treated for 48 h. Distilled water without the addition of NaCl was used to cultivate the control plants. According to the phenotypic variation, 100 mM was the most suitable concentration for the stress treatment. The leaves and roots, as two distinct organs, were selected; each sample included 12 plants. The leaf–stress and root–stress in Longya10 and Fanni were marked as S–Ly–L, S–Ly–R, S–Fn–L, and S–Fn–R, respectively; the parallel controls were marked with M–Ly–L, M–Ly–R, M–Fn–L, and M–Fn–R. We tested the fresh weight of all samples. The analysis of the plant’s physiological parameters was provided by the *Sangon Biotech* technique center (Shanghai, China), including detecting proline, soluble protein, soluble sugar, SOD, POD, CAT, and MDA. All of the physiological parameters assays contained three biological replicates, each of which contained three technical replicates, all of the samples were frozen in liquid N2 and kept at −80 °C until assay.

### 4.2. RNA Extraction, Sequencing, and Mapping

RNA extraction and cDNA library construction methods were used as reported previously [65]. Three biological replicates were analyzed for each sample. These samples were sequenced by Next-Generation Sequencing (NGS) based on the Illumina Novaseq platform. The datasets generated and analyzed in this study are available at [PRJNA860005] https://www.ncbi.nlm.nih.gov/sra/PRJNA860005 (accessed on 20 June 2022). We removed the adaptor, ploy-N, and low-quality reads and obtained >4 Gb clean data with pairs of 150 bp reads of each sample (Table S1). Both Q20 and Q30 were greater than 92%. The flax genome sequence *Lusitatissimum_200_BGIv1.0.fa.gz* and annotation file *Lusitatissimum_200_v1.0.annotation_info.txt* were downloaded from the website (https://data.jgi.doe.gov/refine-download/phytozome?organism=Lusitatissimum&expanded=Phytozome-200, accessed on 13 August 2021). BWA and STAR were used for mapping analysis, and the differentially expressed genes (DEGs) were identified using the DESeq2 R package as in our previous reports [65]. The used criterion was a greater than two-folds change and a significant q value (false discovery rate-adjusted *p* value) of <0.01 to estimate differentially expressed genes [65]. The differentially expressed genes related to hormone metabolism, TFs activity, ionic transport, and epigenetic modification were compared to the annotation file and MapMan software [66].

### 4.3. Gene Ontology (GO) Analysis

GO analysis was performed using the online agriGOv2 platform (http://systemsbiology.cau.edu.cn/agriGOv2/index.php, accessed on 30 August 2021), and the molecular function category of GO terms with an FDR-corrected *p*-value < 0.05 were considered as overrepresented.

### 4.4. qRT-PCR Analysis

To verify the RNA-seq data’s reliability and quantify the expression of AP2-ERF transcription factor in flax under saline stress, the SYBR Green I PCR master mix kit (TaKaRa, Japan) was used in the qRT-PCR reactions, as in our previous reports [67]. The gene-specific primers (Table S7) were downloaded from qPrimerDB [68].

## Data Availability

The datasets generated and analyzed in this study are available at [PRJNA860005] https://www.ncbi.nlm.nih.gov/sra/PRJNA860005 (accessed on 20 June 2022).

## Supplementary Materials

The following supporting information can be downloaded at: https://www.mdpi.com/article/10.3390/plants11152053/s1, Figure S1: The results of five genes expression compared RNA-seq and qRT-PCR in fanni (a) and longya10 (b), respectively; Figure S2: The expression of genes related to photosynthesis-related pathways in leaves of flax under saline stress; Table S1: The overview of RNA-seq data; Table S2: The data of GO analysis; Table S3: The data of phytohormones related genes expression analysis; Table S4: The data of transcription factors related genes expression analysis; Table S5: The data of ionic transport related genes expression analysis. Table S6: The data of epigenetic modification related genes expression analysis; Table S7: The list of primers for qRT-PCR.
