# Supplementary material for: Comprehensive Analysis of Differentially Expressed Genes and Epigenetic Modification-Related Expression Variation Induced by Saline Stress at Seedling Stage in Fiber and Oil Flax, Linum usitatissimum L
Source: Plants (Basel). 2022 Aug 5;11(15):2053. doi: 10.3390/plants11152053 (PMC9370232; doi:10.3390/plants11152053)
Supplement: Supplementary file 1 [file plants-11-02053-s001.zip › SupplementaryMaterials_Legends.pdf]

Supplementary Figure S1: The results of five genes expression compared RNA-seq and qRT-PCR in fanni (a) and longya10 (b), respectively. ∗ $p < 0.05$.

Supplementary Figure S2: The expression of genes related to photosynthesis-related pathways in leaves of flax under saline stress.

Supplementary Table S1： The overview of RNA-seq data.

Supplementary Table S2： The data of GO analysis.

Supplementary Table S3： The data of phytohormones related genes expression analysis.

Supplementary Table S4： The data of transcription factors related genes expression analysis.

Supplementary Table S5： The data of ionic transport related genes expression analysis.

Supplementary Table S6： The data of epigenetic modification related genes expression analysis.

Supplementary Table S7： The list of primers for qRT-PCR.
