# Supplementary figures and images for: Comprehensive Analysis of Differentially Expressed Genes and Epigenetic Modification-Related Expression Variation Induced by Saline Stress at Seedling Stage in Fiber and Oil Flax, Linum usitatissimum L
Source: Plants (Basel). 2022 Aug 5;11(15):2053. doi: 10.3390/plants11152053 (PMC9370232; doi:10.3390/plants11152053)

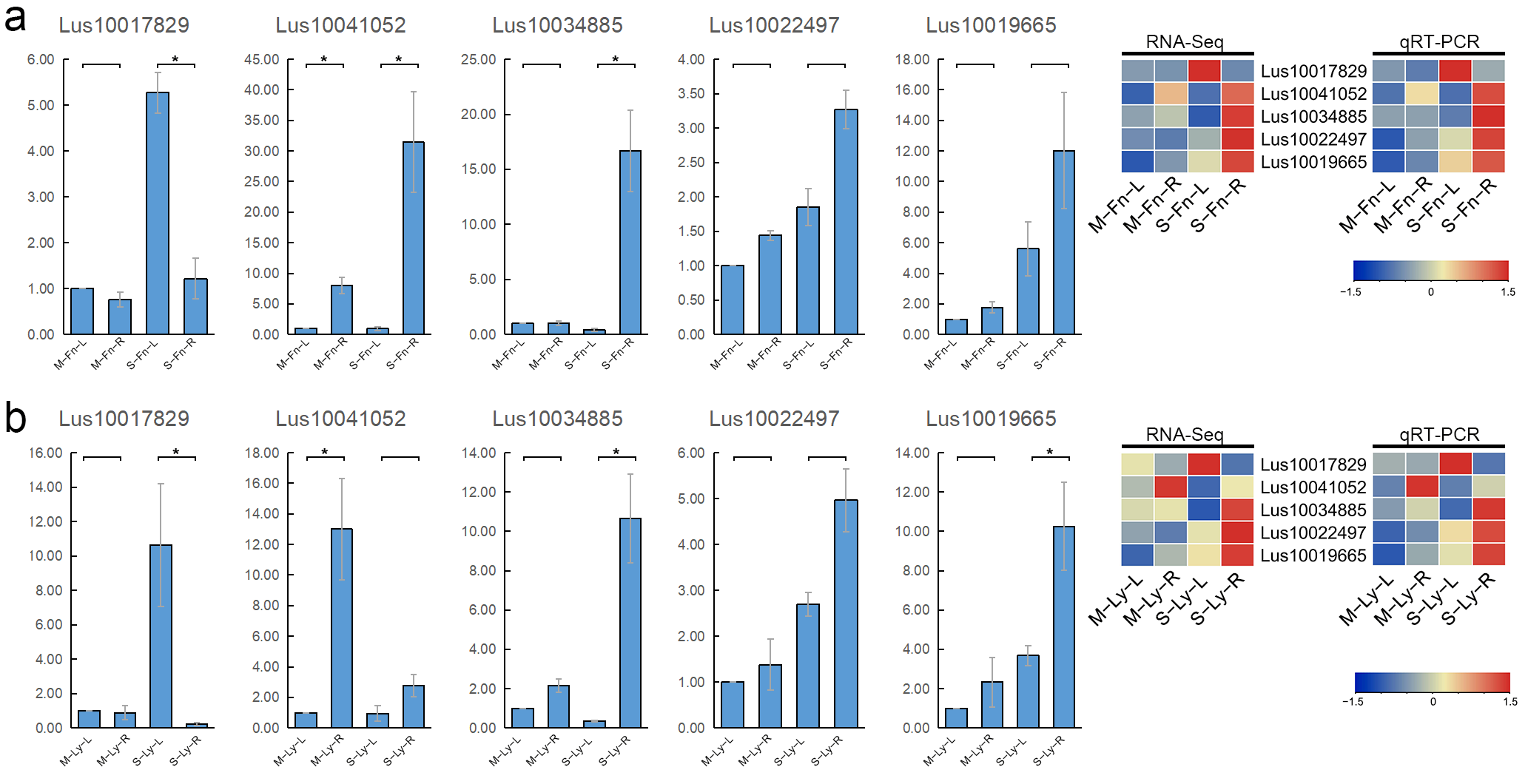

Supplement: Supplementary file 1 [file plants-11-02053-s001.zip › Supplementary Figure S1.tif]

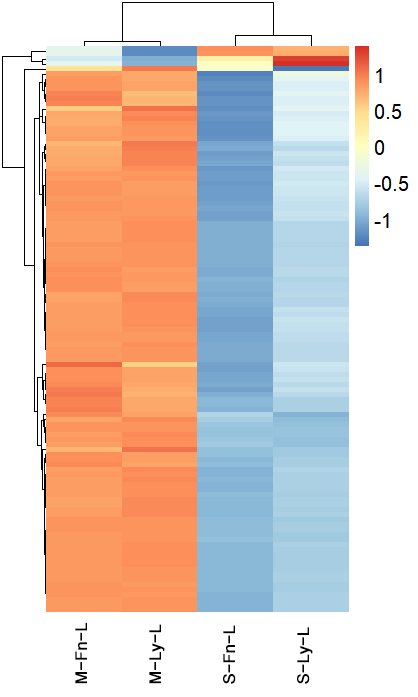

Supplement: Supplementary file 1 [file plants-11-02053-s001.zip › Supplementary Figure S2.tif]
